# TNT‐Induced Phagocytosis: Tunneling Nanotubes Mediate the Transfer of Pro‐Phagocytic Signals From Apoptotic to Viable Cells

**DOI:** 10.1002/jcp.25584

**Published:** 2017-03-31

**Authors:** Margarethe Bittins, Xiang Wang

**Affiliations:** ^1^ Department of BiomedicineUniversity of BergenBergenNorway

## Abstract

The exposure of phosphatidylserine (PS) on the surface membrane of apoptotic cells triggers the recruitment of phagocytic receptors and subsequently results in uptake by phagocytes. Here we describe how apoptotic cells can use intercellular membrane nanotubes to transfer exposed PS to neighboring viable cells, and thus deposit an “eat‐me” tag on the viable cells. Tunneling nanotubes (TNTs) connected UV‐treated apoptotic rat pheochromocytoma PC12 cells with neighboring untreated cells. These TNTs were composed of PS‐exposed plasma membrane and facilitated the transfer of the membrane from apoptotic to viable cells. Other pro‐phagocytic signals, such as oxidized phospholipids and calreticulin, were also transferred to viable cells. In addition, anti‐phagocytic signal CD47 presenting on the plasma membrane of viable cells was masked by the transferred PS‐membrane. Confocal imaging revealed an increase of phagocytosis of viable PC12 cells by murine RAW264.7 macrophages when the viable PC12 cells were cocultured with UV‐treated PC12 cells. Treatment with 50 nM cytochalasin D would abolish TNTs and correspondingly inhibit this phagocytosis of the viable cells. Our study indicates that exposed‐PS membrane is delivered from apoptotic to viable cells through TNTs. This transferred membrane may act as a pro‐phagocytic signal for macrophages to induce phagocytosis of viable cells in a situation where they are in the vicinity of apoptotic cells. J. Cell. Physiol. 232: 2271–2279, 2017. © 2016 The Authors. *Journal of Cellular Physiology* Published by Wiley Periodicals Inc.

**Abbreviations**
AF: Alexa Fluor
CTB: CellTracker Blue CMAC
CTG: CellTracker Green CMFDA
CRL: calreticulin
cytoD: cytochalasin D
OxPL: oxidized phospholipids
PS: phosphatidylserine
TNT: tunneling nanotube
WGA: wheat germ agglutinin

The removal of apoptotic cells in multicellular organisms is critical for development, tissue remodeling, and maintenance of homeostasis. The recognition and engulfment of dead cells by phagocytes is guided by a wide variety of cell surface receptors and soluble bridging molecules (Ravichandran, 2011). One of the main “eat‐me” signals is the exposure of phosphatidylserine (PS) on the outer leaflet of the membrane of apoptotic cells when the membrane loses phospholipid asymmetry (Fadok et al., 2001). Moreover, the presence of calreticulin and oxidation‐specific epitopes on the surface of apoptotic cells also serve as crucial recognition and clearance ligands (Chang et al., 1999; Gardai et al., 2005). Meanwhile, apoptotic cells normally lose “don't eat‐me” signals on plasma membrane, such as CD47 (an integrin‐associated protein) that otherwise interacts with SIRPα on the efferocyte (Gardai et al., 2005). Besides endogenous generation of signals, exogenous acquisition of signals can also induce phagocytosis. For instance, addition of liposomes containing PS to viable HL‐60 cells results in a transient elevation of PS on the surface of the cells, which promotes their phagocytosis by macrophages (Fadok et al., 2001). A similar result was shown by Shurin et al. (2009): exogenous labeling of viable tumor cells with PS‐liposomes could result in engulfment of the tumor cells by dendritic cells. These findings suggest that exogenous PS present on viable cells can promote recognition and phagocytosis of viable cells by phagocytes.

In the last decade, a new cell‐to‐cell nano‐scaled membrane connection named tunneling nanotube (TNT) or membrane nanotube has been discovered (Davis and Sowinski, 2008). These thin intercellular membrane channels are about 50–200 nm in diameter and contain F‐actin as the major cytoskeletal component (Rustom et al., 2004). To date, TNTs have been found in numerous cell types such as fibroblasts, epithelial cells and immune cells (Austefjord et al., 2014), as well as in primary cells including neurons and astrocytes (Wang et al., 2012). In vivo observation has proven the presence of TNT‐like structures in different tissues, such as mouse cornea (Chinnery et al., 2008; Seyed‐Razavi et al., 2013), chicken and zebrafish embryo (Caneparo et al., 2011; McKinney et al., 2011). Functional analysis revealed that TNTs facilitate intercellular transfer of depolarization signals and a range of cellular compounds including calcium, membrane protein, cellular organelles, and vesicles (Wang et al., 2010; Abounit and Zurzolo, 2012; Wang and Gerdes, 2012; Burtey et al., 2015). Furthermore, pathogens, such as HIV‐1 and prion proteins, have been shown to use nanotubular structures to spread from infected to healthy cells (Sowinski et al., 2008; Gousset et al., 2009).

TNTs are also involved in the modulation of cell death. It has been shown that they participate in the rescue of injured cells via delivery of organelles or calcium signal from healthy cells (Cselenyak et al., 2010; Naphade et al., 2015; Osswald et al., 2015; Wang and Gerdes, 2015). In contrast, Chauveau et al. (2010) discovered that TNTs could aid the lysis of distant cells either directly or by moving target cells to natural killer cells for lysis at a conventional immune synapse. Another study reported that the death signal Fas ligand was transferred between T lymphocytes via TNTs and induced cell death (Arkwright et al., 2010). Here, we found that apoptotic PC12 cells transfer PS‐positive membrane (PS‐membrane) to neighboring viable cells through TNTs. Notably, the phagocytosis of viable cells by macrophages increased when apoptotic cells were present, but only under conditions that allowed for the formation of TNTs. Our findings suggest that TNTs are a means of transferring PS‐membrane as an “eat‐me” signal from dying to living cells, and thus induce phagocytosis of previously healthy cells.

## Materials and Methods

### Cell culture and induction of apoptosis

PC12 cells (rat pheochromocytoma cells, clone 251) were cultured in DMEM (Life Technologies, Gaithersburg, MD) supplemented with 10% fetal calf serum (PAA Laboratories GmbH, Pasching, Austria) and 5% horse serum (PAA Laboratories) at 37°C in a humidified atmosphere containing 10% CO_2_ (Rustom et al., 2004). Only passage 15–20 was used in experiments. RAW264.7 cells (TIB‐71™) were obtained from the American Type Culture Collection and cultured in DMEM containing 10% fetal calf serum in a 5% CO_2_ incubator.

For the coculture experiments, PC12 cells (6 × 10^4^ cells/well) were placed in µ‐Slide 8 well chambers (Ibidi GmbH, Martinsried, Germany) coated with poly‐L‐lysine (0.1 mg/ml, Sigma–Aldrich Corp., St. Louis, MO) and cultured overnight in a 10% CO_2_ incubator. Then cells were stained with 20 µM CellTracker Blue CMAC (CTB) or 5 µM CellTracker Green CMFDA (CTG, Invitrogen, Carlsbad, CA) at 37°C for 45 min, before medium was replaced with 300 µl fresh medium. After apoptosis inducing treatment, CellTracker‐labeled cells were cocultured with untreated, unlabeled PC12 cells (6 × 10^4^ cells/well) in the 10% CO_2_ incubator for 24 h.

To induce apoptosis, PC12 cells were irradiated by UV‐C light (TUV G15T8, Philips, Eindhoven, Netherland) for 150 sec 24 h later, 54.9 ± 3% of the cells were PS‐positive (apoptotic). Lower dose UV treatment (90 sec) induced apoptosis in 12.9 ± 1.3% of the cells, which form fewer PS‐TNTs. In higher dose (>180 sec), cells enter into necrosis and detach from the substrate after 24 h.

### Live imaging of the formation of PS‐TNT

Cocultures of UV‐treated CTB‐PC12 cells and untreated/unlabeled PC12 cells were stained with annexin V‐Alexa Fluor (AF) 488 (Invitrogen) for 5 min and imaged using a Leica SP5 laser scanning confocal microscope (Leica Microsystems GmbH, Mannheim, Germany) equipped with a 40× oil immersion objective (HCX PL APO CS 40.0 × 1.25 OIL UV), a 37°C and humidified 10% CO_2_ chamber. A motorized stage allowed multi‐position imaging of several potential donor‐acceptor pairs simultaneously. Prior to the time‐lapse, a single stack was taken with the blue laser (405 nm) to show the position of the CTB‐labeled apoptotic cells. Live imaging was performed by scanning 14 z‐sections at 1.5 μm distance for 1 h at 1 min intervals with resonant scan mode at 8000 Hz.

### Immunefluorescence and membrane staining

Cocultures of UV‐treated CTB‐PC12 and untreated/unlabeled PC12 cells in µ‐Slide 8 well chambers were fixed with 4% paraformaldehyde/4% sucrose in PBS 24 h after plating. Immunefluorescence staining was performed without permeabilization by using primary antibody: rabbit polyclonal anti‐calreticulin (Abcam, Cambridge, UK), mouse monoclonal Anti‐oxidized phospholipid (Avanti, Alabaster, AL), rabbit polyclonal anti‐CD47 (Abcam), and secondary antibody: goat anti‐mouse‐AF564, goat anti‐rabbit‐AF647 (Invitrogen). Wheat germ agglutinin (WGA)‐AF594 or WGA‐AF633 (Invitrogen) was used to stain plasma membrane. F‐actin was labeled with phalloidin‐AF488 (Invitrogen) after permeabilization. Microtubules were stained using mouse monoclonal anti‐α‐tubulin (Sigma–Aldrich). For PS staining, cells were incubated with annexin V‐AF488 for 30 min before fixation. All solutions contained 2 mM Ca^2+^ in the experiment of annexin V‐AF488 staining. 3D‐confocal imaging was performed on the Leica TCS SP5 confocal microscope equipped with the resonant scanner and a HCX PL APO CS 63 × /1.40 NA or the 40 × /1.25 NA oil‐immersion objective.

### Phagocytosis assay by confocal imaging

CTG‐labeled target PC12 cells (CTG‐PC12, 6 × 10^4^ cells/well) were cocultured with unstained UV‐treated or untreated PC12 cells (UV‐PC12, 6 × 10^4^ cells/well) in µ‐Slide 8 well chambers for 24 h. Then Vybrant DiD (Invitrogen) stained RAW264.7 cells (DiD‐RAW264.7, 3 × 10^4^ cells/well) were added into the PC12 cell coculture. In control condition, UV‐treated CTG‐PC12 cells (6 × 10^4^ cells/well) were cocultured with DiD‐RAW264.7 cells (3 × 10^4^ cells/well). After 24 h of coculture, cells were imaged randomly by using the Leica TCS SP5 confocal microscope with the resonant scanner and the 40 × /1.25 NA oil‐immersion objective. In each condition, 10 3D‐stacks containing an average of 480 RAW264.7 cells were analyzed. For image analysis, a threshold value (background fluorescence of green channel) was subtracted from the 3D fluorescence images in each experiment. The phagocytosis of PC12 cells by RAW264.7 cells was expressed as the number of DiD‐RAW264.7 cells containing CTG signals in the maximum projection of the 3D images.

### Caspase‐3 activity

The activation of caspase‐3 was measured using Image‐iT LIVE Green Caspase‐3/7 Detection Kit (Invitrogen). Briefly, CTB‐labeled (UV‐treated) and non‐labeled PC12 cells were cocultured for 24 h and then incubated with FLICA solution for 90 min and annexin V‐AF568 (1:125) for 45 min. Then cells were fixed and washed. The images were obtained with the Leica TCS SP5 confocal microscope equipped with the resonant scanner and the 40 × /1.25 NA oil‐immersion objective with excitation wavelength of 405 nm (CTB), 488 nm (FLICA), and 561 nm (annexin V‐AF568).

To quantify the fluorescence intensity of FLICA (caspase‐3 activity) in cells, all 3D confocal imaging were performed with fixed settings in each experiment. The collection of maximum projections of 3D imagings, the selection of ROIs and the measurement of the average fluorescence intensity of FLICA were performed by using the Leica LAS AF Lite software (Leica Microsystems GmbH). The relative fluorescence intensity (RFI) value of FLICA per cell was calculated by dividing the mean intensity of PS‐TNT connected cells by the mean intensity of control viable cells without TNT connection in the same image. The RFI value of FLICA in control viable cells was normalized to one.

### Quantification of TNTs

Cocultures of CTB‐labeled and unlabeled PC12 cells were treated with different concentrations of cytochalasin D (Sigma–Aldrich) for 24 h and stained with WGA‐AF488 (Invitrogen) for 5 min. Then live‐cell imaging was performed by using a Zeiss Axiovert 200 M fluorescence microscope (Carl Zeiss, Jena, Germany) equipped with a 63 × /1.40 NA oil‐immersion objective and a DAPI/FITC/TRITC filter set. For each condition, stacks of 20 images from the bottom to the top of cells were acquired at 400 nm (CTB) and at 488 nm (WGA‐AF488) within 20 min 20 stacks containing at least 350 cells were obtained in each condition. The numbers of TNTs in the CTB‐labeled and the unlabeled populations were counted and expressed as number of TNTs per 100 cells.

For counting of PS‐positive TNTs, UV‐treated (CTB‐labeled), and untreated PC12 cells were cocultured for 24 h in the presence or absence of 50 nM cytoD. Then cells were stained with annexin V‐AF488 for 45 min, followed by live imaging using the Leica TCS SP5 confocal microscope equipped with the resonant scanner and the HCX PL APO CS 40 × /1.25 NA oil‐immersion objective. In each condition, 20 3D‐images were obtained to ensure that more than 1700 cells were acquired for the counting. The numbers of PS‐TNT between the CTB‐labeled and the unlabeled cells were counted and expressed as number of TNTs per 100 PS and CTB double positive cells.

### Phagocytosis assay by flow cytometry

CTG‐labeled target PC12 cells (CTG‐PC12, 7.5 × 10^5^ cells) were cocultured with UV‐treated unstained PC12 cells (7.5 × 10^5^ cells) in a 3.5 cm petri dish for 24 h. Then DiD‐labeled RAW264.7 cells (5 × 10^5^ cells) were added into the PC12 cell coculture and incubated for 24 h. Fifty nanomolar cytochalasin D was present in the cell culture medium, where indicated. Cells were harvested and suspended in cold PBS for flow cytometry analysis. Data acquisition was performed on an Accuri C6 flow cytometer using Cflow plus software (BD Biosciences, San Jose, CA). For each condition, 40,000 cells were analyzed. DiD‐labeled RAW264.7 and CTG‐labeled PC12 control populations under the same experimental condition were measured for gating. Phagocytosis was evaluated based on the percentage of CTG/DiD positive RAW264.7 to total DiD‐RAW264.7 cells.

### Statistical analyses and 3D reconstruction

In all cases, at least three independent experiments were performed. Values are expressed as means ± s.e.m. Data were analyzed with Student's two‐tailed *t* test (Microsoft Excel, WA). Differences were considered significant at *P* < 0.05. The 3D reconstructions were generated by Imaris 3D software (Bitplane AG, Zürich, Switzerland).

## Results

### Formation of PS‐positive TNTs and transfer of PS‐positive membrane

**Figure 1:**
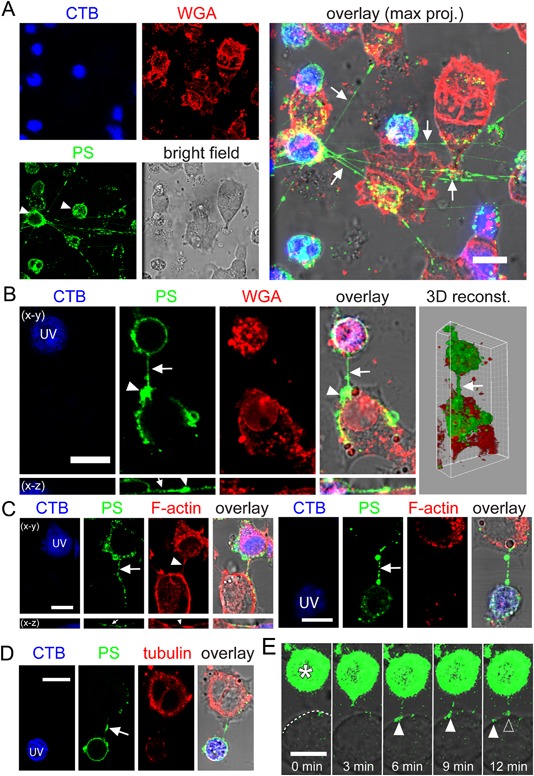
Transfer of PS‐exposed plasma membrane from apoptotic to viable PC12 cells via PS‐TNTs. CTB‐labeled PC12 cells were treated with UV and cocultured with untreated PC12 cells for 24 h. (A) The presence of PS‐positive TNT (arrows) between apoptotic (arrowheads) and viable PC12 cells. The cells were labeled with annexin V‐AF488 (PS) and WGA‐AF594 (WGA). (B) PS‐exposed membrane was transferred through a PS‐TNT (arrow) from a UV‐treated apoptotic cell (*UV*) to a viable cell and accumulated on the contact site (arrowhead). The 3D reconstruction image shows that the PS‐membrane coated a large area of surface of the receiving cell. Cells were labeled as in (A). (C) F‐actin (arrowheads) was present (left) or absent (right) in PS‐TNTs (arrows). The cells were labeled with annexin V‐AF488 (PS) and phalloidin‐AF594 (F‐actin). (D) PS‐TNTs (arrow) did not contain microtubules. The cells were labeled with annexin V‐AF488 (PS) and immunostained with anti‐α‐tubulin antibody (tubulin). (E) The formation of PS‐TNT and transfer of PS‐membrane to viable cell. Live‐cell imaging from a 60 min time‐lapse recording shows a UV‐treated apoptotic cell (asterisk) that sends a PS‐TNT to a neighboring cell (dashed line). The PS at the tip of the TNT separates from it and moves along the surface of the acceptor cell (arrowheads). Scale bars, 10 μm.

To investigate whether PS can be transferred via TNTs from apoptotic to viable cells, we selected PC12 cells because they form numerous TNTs that can facilitate intercellular transfer of vesicles and membrane‐associated proteins (Rustom et al., 2004). We used a coculture system where PC12 cells labeled with CellTracker Blue (CTB) were treated with UV‐light to induce apoptosis and cocultured with unlabeled and untreated PC12 cells. After 24 h, cocultures were stained with annexin V‐AF488 to label exposure of PS on membrane and WGA‐AF594 to label glycoproteins as a plasma membrane marker. Confocal imaging showed that apoptotic cells exposed PS and formed PS‐positive TNTs (PS‐TNTs) contacting viable cells nearby (Fig. 1A). Notably, PS was often found on the surface of viable cells that were connected with apoptotic cells via PS‐TNTs, suggesting transfer of PS‐membrane from the apoptotic to viable cells (Fig. 1B). The 3D reconstruction showed that the transferred PS‐membrane covered a large area of the viable cell's surface. In contrast to “normal” TNTs that can be labeled with WGA and contain F‐actin (Rustom et al., 2004), the PS‐TNTs formed by apoptotic cells were devoid of WGA label (Fig. 1B), and only five out of 33 contained F‐actin (Fig. 1C). This is consistent with the fact that WGA‐stained glycoproteins and F‐actin are cellular components that disappear from dead or dying cells (Brown et al., 1997; Sarter et al., 2007). Immunostaining revealed that none of the PS‐TNTs observed contained microtubules (n = 10, Fig. 1D), indicating that they were also different from the microtubule‐positive TNTs in the stressed PC12 cells we described earlier (Wang and Gerdes, 2015).

Live imaging of the apoptotic and viable cell pairs with short distance showed the formation of a PS‐TNT, originating from a short PS‐positive protrusion from an apoptotic cell. The protrusion elongated, moved towards and finally contacted the neighboring viable cell within about two minutes (Fig. 1E). PS‐membrane from the tip of the PS‐TNT was then transferred to the surface of the viable cell. The patch of PS‐membrane could be distinguished as a discrete entity ten minutes after formation of the PS‐TNT. When the TNT broke, PS‐membrane remained on the previously unlabeled healthy cell and was not engulfed for over an hour after staining. In four instances, we observed this type of transfer on TNTs that already existed at the start of imaging (four independent experiments, not shown).

### Transfer of other pro‐phagocytic signals and masking of anti‐phagocytic signal

**Figure 2:**
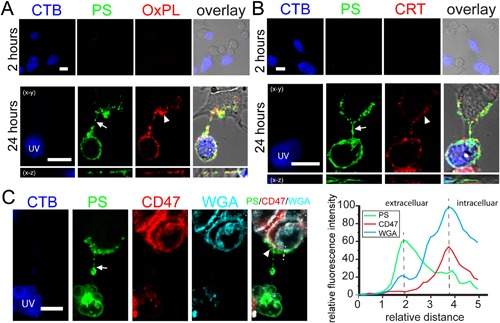
Transfer of other pro‐phagocytic signals and masking of anti‐phagocytic signal on viable PC12 cells. CTB‐labeled PC12 cells were treated with UV light and cocultured with untreated cells for 2 or 24 h. PS‐membrane was stained with annexinV‐AF488 (PS). (A, B) OxPL and CRT were present in PS‐TNTs (arrows) and TNT‐connected viable cells (arrowheads) after 24 h coculture. Note that PS, OxPL and CRT were not exposed on the surface of viable cells after 2 h coculture. Cocultures were immunostained with anti‐OxPL or anti‐CRT respectively. (C) CD47 (arrowheads) on the membrane of viable cell was coated by transferred PS‐membrane (arrow). Cocultures were immunostained with anti‐CD47 and WGA‐AF633 (WGA). The intensity profile along the dashed line demonstrates the different distribution of PS (green), CD47 (cyan) and WGA (red) signals. Scale bars, 10 μm.

PS acts as a pro‐phagocytic signal in conjunction with other “eat‐me” signals (Ravichandran, 2011). For example, treatment with UV‐light causes the exposure of calreticulin (CRT) on the cell surface, and oxidizes phospholipids that serve as additional pro‐phagocytic signals (Chang et al., 1999; Gardai et al., 2005; Obeid et al., 2007). We therefore probed for the presence of oxidized phospholipid (OxPL) and CRT on apoptotic cells, and tested whether they could be transferred to viable cells through PS‐TNTs. Cocultures were fixed without permeabilization, and immunostained with antibodies against OxPL and CRT separately. As expected, there was no surface labeling of PS, OxPL, or CRT on viable or UV‐treated cells 2 h after UV‐treatment and coculture, and it was strong on apoptotic cells after 24 h (Fig. 2A and B). Importantly, OxPL and CRT were also found on viable cells that were connected with apoptotic cells via PS‐TNTs (Fig. 2A and B). Moreover, they colocalized with PS on PS‐TNT and on the viable cells, suggesting that whole apoptotic membrane batches containing PS, CRT, and OxPL have been transferred.

Most viable cells express the surface antigen CD47 that is considered an anti‐phagocytic “don't eat‐me” signal (Gardai et al., 2005). As we saw that the surface of viable cells could be partially coated with transferred PS‐membrane, we asked whether CD47 was expressed, and where it was localized in these viable cells. Immunostaining showed that, as expected, CD47 was found on viable, but not on dying PC12 cells (Fig. 2C). Interestingly, part of CD47‐positive plasma membrane on the viable cell was coated by transferred PS‐membrane. Intensity profile analysis of the fluorescence signal shows that the transferred membrane outside was labeled with annexin V and the plasma membrane inside was labeled with WGA and anti‐CD47 (Fig. 2C), suggesting two membranes were parallel to each other and may not have fused. Instead, it seems like the PS‐membrane formed a cup‐shaped structure on top of the viable cell. Overall, the transferred PS‐membrane could act as a dominant pro‐phagocytic signal, and suppress recognition of the anti‐phagocytic signal CD47.

### Phagocytosis of viable PC12 cells by RAW264.7 macrophages

**Figure 3:**
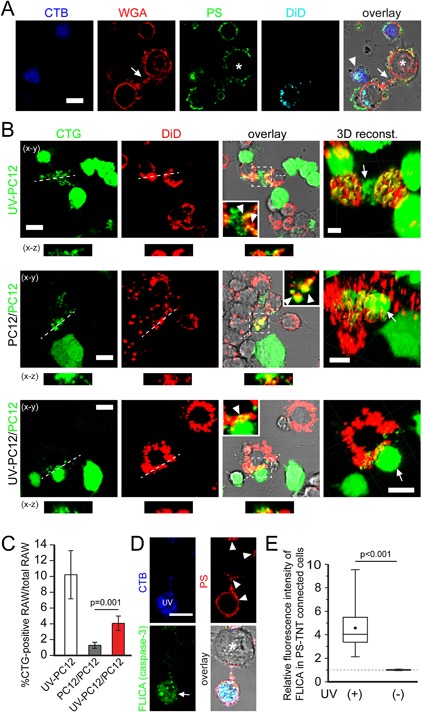
Phagocytosis of viable PC12 cells by RAW264.7 cells increased when the viable cells were cocultured with apoptotic PC12 cells. (A) An intracellular bridge (arrow) formed between a RAW264.7 cell and an untreated but PS‐exposing PC12 cell (asterisk). UV‐treated PC12 cells (CTG) were cocultured with untreated PC12 cells (unlabeled) for 24 h, then cocultured with RAW264.7 macrophages (DiD) for 6 h. The cells were stained with annexin V AF‐488 (PS) and WGA‐AF594 (WGA). (B) The phagocytosis of PC12 cells by RAW264.7 cells. Top: UV‐treated PC12 cells (CTG) were cocultured with RAW264.7 cells (DiD) for 24 h. Middle: untreated PC12 cells (unlabeled) were cocultured with untreated PC12 cells (CTG) for 24 h. Then cells were cocultured with RAW264.7 cells (DiD) for 24 h. Bottom: UV‐treated PC12 cells (unlabeled) were cocultured with untreated PC12 cells (CTG) for 24 h. Then cells were cocultured with RAW264.7 cells (DiD) for 24 h. Overlay in the magnified confocal images and 3D reconstruction (dark yellow) illustrate how macrophages contact CTG‐labeled cells (arrows), and engulf CTG particles (arrowheads). (C) Quantification of phagocytosis assay in (B). n = 4 independent experiments. (D) Caspase‐3 is not active in viable PC12 cells coated with PS‐membrane. CTB‐PC12 cells were treated with UV and cocultured with untreated PC12 cells for 24 h. Then cells were stained with annexin V‐AF568 (PS) and Image‐iT LIVE Green Caspase‐3/7 Detection Kit (FLICA). Confocal imaging shows that both UV‐treated and viable cells exposed PS (arrowheads) but only the dead cells showed caspase‐3/7 activity (arrow). (E) Quantification of caspase‐3 activation in PS‐TNT connected cell pairs (n = 31 pairs). The relative fluorescence intensity of FLICA (caspase‐3) was measured and calculated as described in Materials and Methods. The mean value is represented by squares and the median by horizontal lines; the 75th and 25th centiles are at the top and bottom of the boxes, respectively. Scale bars, 10 μm.

To explore whether transferred PS‐membrane could act as a signal to induce phagocytosis of viable cells by macrophages, we established a triple‐culture system composed of apoptotic and viable PC12 cells and RAW264.7 macrophages. RAW264.7 cells would make contact with both apoptotic cells (arrowhead, Fig. 3A) and viable cells that were covered with PS‐exposed membrane (asterisk, Fig. 3A). To measure phagocytosis in this system, we labeled either UV‐treated or viable PC12 cells with CellTracker Green (CTG) and quantified the colocalization with DiD‐labeled RAW264.7 macrophages. In the positive control, where UV‐treated PC12 cells (54.9 ± 3% of the cells underwent apoptosis after 24 h) were cocultured with RAW264.7 cells, 10.2 ± 3.1% of macrophages would readily contact and ingest apoptotic PC12 cells (top panel, Fig. 3B and C). We also found that very few macrophages would uptake untreated PC12 cells (middle panel, Fig. 3B), that probably entered cell death spontaneously during culture. We did not observe RAW264.7 cells engulfed PC12 cells in their entirety, possible owing to a different size between them. Interestingly, untreated cells were much more likely to be phagocytized by macrophages (bottom panel, Fig. 3B) in the presence of UV‐treated PC12 cells compared to the condition without UV‐treated PC12 cells (4.1 ± 1% and 1.3 ± 0.4%, respectively, Fig. 3C).

The observed increase in phagocytosis, as well as the PS‐exposure, are indications that the cells in our coculture system may not have been viable, but had started to undergo apoptosis themselves. To test this possibility, we probed for the activation of caspase‐3, a key enzyme in the pro‐apoptotic proteolytic signaling cascade that is required for exposure of PS (Suzuki et al., 2013; Segawa et al., 2014). Levels of active caspase‐3 were high in the UV‐treated cells, consistent with induction of apoptosis by the UV‐light (Fig. 3D and E). However, activity in the untreated cell was negligible, despite the presence of PS‐exposed membrane and a PS‐TNT connection with an apoptotic cell (Fig. 3D and E). Thus, phagocytosis, as well as PS‐exposure on viable cells, cannot be explained by the induction of apoptosis in these cells. Rather, it indicates alternative ways of acquiring PS‐membrane as a pro‐phagocytic signal.

### Phagocytosis of viable cells is dependent on TNTs

**Figure 4:**
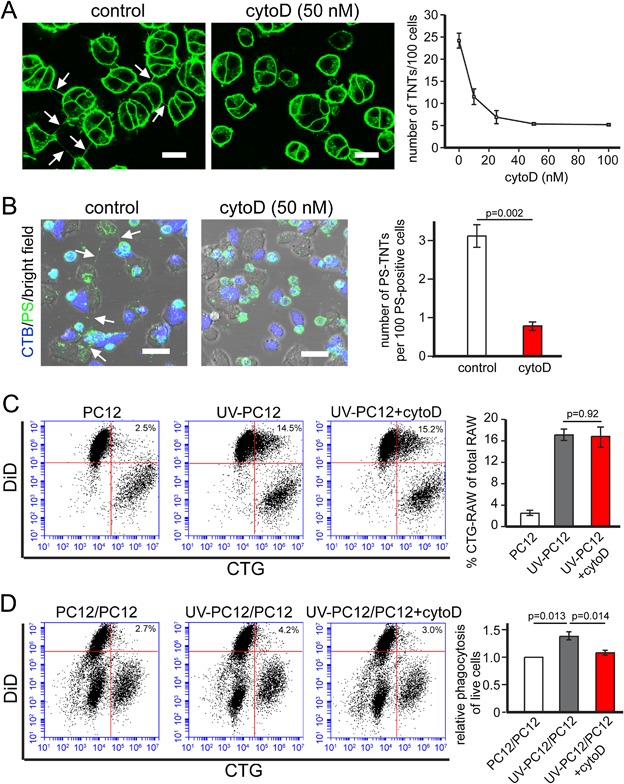
Inhibition of TNT formation deceased phagocytosis of viable PC12 cells. (A) Dose‐dependent inhibition of TNT number in PC12 cells after 24 h treatment with cytoD. Scale bars, 20 μm. (B) PS‐positive TNTs in PC12 cells were abolished by 24 h treatment of 50 nM cytoD. Scale bars, 30 μm. (C) 50 nM cytoD did not inhibit phagocytosis of apoptotic PC12 cells. DiD‐RAW264.7 cells were cocultured with CTG‐stained untreated or UV‐treated PC12 cells in the absence and presence of 50 nM cytoD for 24 h, resp. Phagocytosis was measured by flow cytometry, and expressed as the percentage of CTG/DiD double‐positive RAW264.7 cells as a subpopulation of all DiD‐positive RAW264.7 cells. (D) Phagocytosis of viable PC12 cells was inhibited by 50 nM cytoD. CTG‐PC12 cells were cocultured with unlabeled UV‐treated or untreated PC12 cells for 24 h. Then DiD‐RAW264.7 cells were added and cultured for 24 h in the presence or absence of 50 nM cytoD. The phagocytosis of CTG‐PC12 cells was measured by flow cytometry and expressed as relative phagocytosis after normalization with control (n = 4).

To further study whether TNTs were responsible for the phagocytosis of viable cells, we tested if removal of TNTs would prevent this phenomenon. Treatment with 350 nM cytochalasin B is known to prevent TNT formation in PC12 cells (Bukoreshtliev et al., 2009). However, it also inhibited phagocytosis of apoptotic PC12 cells in our coculture system (data not shown). As an alternative we tested another F‐actin depolymerizing agent, cytochalasin D (cytoD), since several studies have previously shown an inhibitory effect on TNT formation (Gurke et al., 2008; Wang et al., 2011; Vallabhaneni et al., 2012). Here, cytoD also inhibited the formation of TNTs in a dose‐dependent manner (n = 3, Fig. 4A) and significantly reduced the number of PS‐TNTs at 50 nM (n = 3, Fig. 4B). At this concentration, cytoD did not influence the phagocytic uptake of apoptotic PC12 cells by RAW264.7 cells (n = 3, Fig. 4C). Then, we investigated the phagocytosis of viable PC12 cells in the same triple‐cultures as before, but used flow‐cytometry for quantification. Viable PC12 cells were labeled with CTG, and cocultured with DiD‐labeled macrophages in the presence or absence of UV‐treated PC12 cells. Untreated PC12 cells were phagocytized at baseline levels (left scatter, Fig. 4D). In accordance with the imaging results presented above (Fig. 3C), phagocytosis of untreated cells was significantly increased, when apoptotic cells were present in the culture dish (middle scatter, Fig. 4D). Notably, this increase was abolished by the presence of 50 nM cytoD (right scatter, Fig. 4D). Taken together, these results indicate that cytoD has no effect on the phagocytosis of apoptotic cells, but it prevents the increase in phagocytosis of viable cells caused by transfer of PS‐membrane.

## Discussion

We report here a novel type of TNT originating from caspase‐3 activated and PS‐exposing apoptotic cells. These TNTs are enriched with PS, but do not contain glycoproteins and rarely F‐actin inside. They arise from filopodia‐like precursors by movement toward non‐apoptotic neighboring cells. Moreover, PS‐TNTs can be abolished by cytoD, suggesting that the formation of PS‐TNTs is based on filopodial interplay. Importantly, we show that PS‐membrane can be transferred in the anterograde direction along PS‐TNTs, from donor/apoptotic to acceptor/viable cells. Although viable cells are known to receive PS‐positive apoptotic bodies and blebs released by apoptotic cells, the PS is then internalized by engulfment of these membrane vesicles (Bergsmedh et al., 2001; Poon et al., 2014). In our live imaging experiments, we did not observe any intracellular PS signal up to 1 h after the staining procedure. This indicates that PS that was transferred by TNTs remains on the surface of the viable cells, thus increasing the chance of recognition by phagocytes.

Phagocytes are attracted to their target cells by chemoattractants, caspase‐dependent chemotactic factors that are secreted by the apoptotic cells (Lauber et al., 2003; Elliott et al., 2009). Our results, however, show that PS‐exposed viable cells did not contain active caspase‐3. They are therefore unlikely to release chemoattractants themselves. Rather, it is possible that macrophages are attracted to the nearby apoptotic cells, which brings them in close physical contact to the PS‐exposed viable cells, and thus allowing for recognition of the “eat‐me” signals on the cell surface of the viable cells. Exposure of PS is critical for macrophage discrimination between apoptotic and non‐apoptotic cells (Borisenko et al., 2003). However, increasing evidence indicates that PS externalization is a necessary but insufficient prerequisite for phagocytosis. Our immunolabeling studies demonstrate the co‐transfer of calreticulin and oxidized phospholipids with PS, which provides viable cells with complete pro‐phagocytic signals. In addition, masking CD47 may shield the anti‐phagocytic signal and promote phagocytosis. Therefore, the appearance of “eat‐me” signals together with the disappearance of “don't eat‐me” signals on viable PC12 cells may determine the recruitment of phagocytic receptors and subsequent phagocytic clearance.

While previous work has focused on the effects of apoptotic cells on the growth and differentiation of neighboring cells (Gregory and Pound, 2010), our data suggest that apoptotic cells can bring death to neighboring viable cells by sending pro‐phagocytic signals through TNTs. Brown and Neher (2012) introduced the term “phagoptosis” to describe cell death of viable cells by phagocytosis. It is a phenomenon that has been found in several physiological processes, such as clearance of PS‐exposed erythrocytes and viable neutrophils. Here, we postulate that phagoptosis may also be caused by obtaining exogenous pro‐phagocytic signals via TNTs. We have observed that apoptotic breast cancer cells (MCF‐7 and MDA‐MB‐231) form PS‐TNTs with viable cells (data not shown). In this context, apoptotic tumor cells may produce a bystander effect to other drug‐resistant tumor cells by mobilizing phagocytes during cancer treatment. Moreover, the transfer of PS to surrounding non‐apoptotic cells may amplify the anti‐inflammatory effect by stimulating macrophages to produce more cytokines and chemokines (Huynh et al., 2002). Therefore, TNT‐dependent transfer of pro‐phagocytic signals may become a potential target in the treatment of disease.
